# Supplementary material for: Lateral line ablation by ototoxic compounds results in distinct rheotaxis profiles in larval zebrafish
Source: Commun Biol. 2023 Jan 21;6:84. doi: 10.1038/s42003-023-04449-2 (PMC9867717; doi:10.1038/s42003-023-04449-2)
Supplement: Supplementary file 2 — Supplemental Material [file 42003_2023_4449_MOESM2_ESM.pdf]

Lateral Line Ablation by Ototoxic Compounds Results in Distinct Rheotaxis Profiles in Larval Zebrafish

Kyle C Newton[1]*, Dovi Kacev[2], Simon R O Nilsson[3], Allison L. Saettele[1], Sam A Golden[3], and Lavinia Sheets[1,4]*

[1] Department of Otolaryngology, Washington University School of Medicine, St. Louis, MO, USA

[2] Scripps Institution of Oceanography, University of California San Diego, La Jolla, CA, USA

[3] Department of Biological Structure, University of Washington, Seattle, WA, USA

[4] Department of Developmental Biology, Washington University School of Medicine, St. Louis, MO, USA

SUPPLEMENTARY INFORMATION

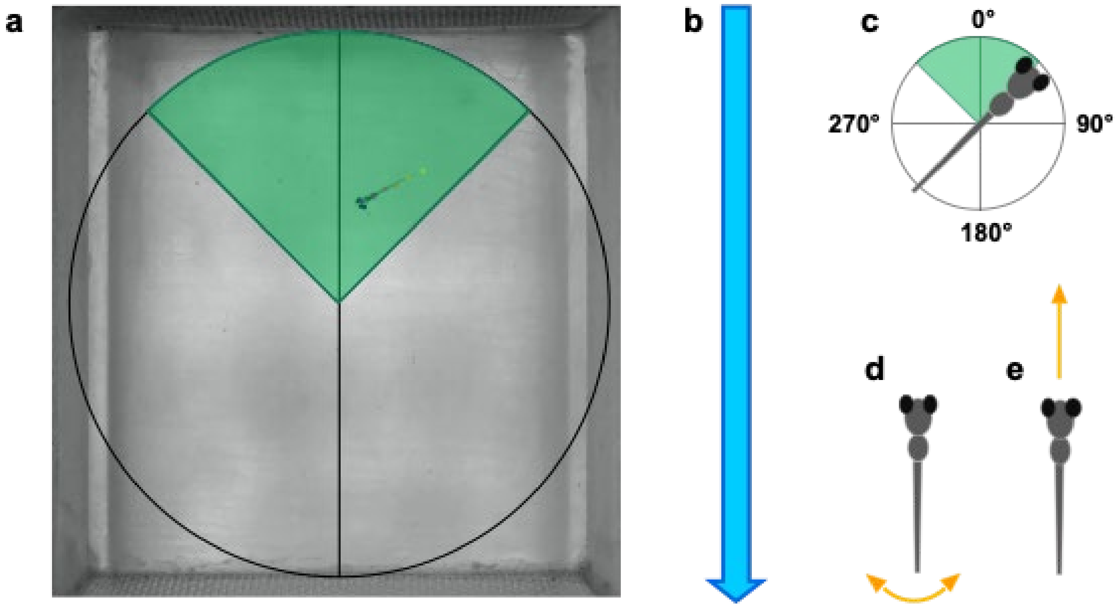

Supplementary Figure 1. Definition of positive rheotaxis behavior. a) Larval zebrafish in the microflume arena performing rheotaxis under flow as defined by multiple conditions, including b) the water flow stimulus was on; c) the fish body angle was oriented to 0° ± 45° (green shaded wedge); d) the tail moved laterally every 100 ms; and e) the body of the fish had forward translation every 100 ms. Note that conditions (d) and (e) were used to discriminate between fish displaying positive rheotaxis and those passively drifting backward with body angles of 0° ± 45°.

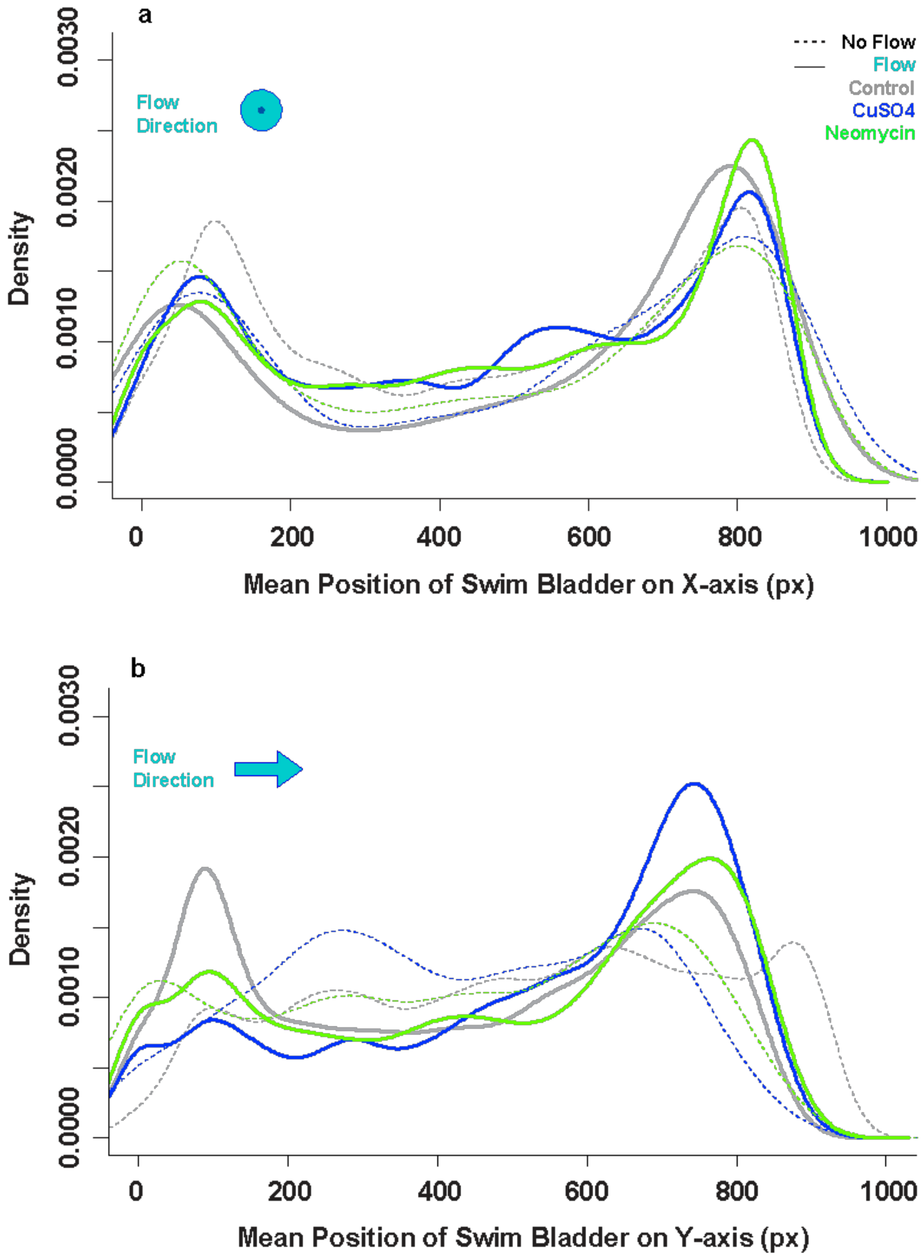

Supplementary Figure 2. Intact lateral line allowed fish to hold their position near the source of the flow. a) The total spatial use of the arena in the X- dimension (left to right) does not differ among treatments (gray = control, blue = $CuSO_4$, green = neomycin) or flow conditions (none = dotted lines, flow = solid lines). All fish preferred to occupy the right versus the left side of the arena. b) in the Y- dimension (front to back) under no flow conditions, the $CuSO_4$ treated fish occupied the center of the arena more than the control or neomycin treated fish. However, under flow conditions, the lateral line intact (control; gray solid line) fish occupied the front of the arena, whereas lesioned fish (blue and green solids lines) predominantly occupied the back of the arena.

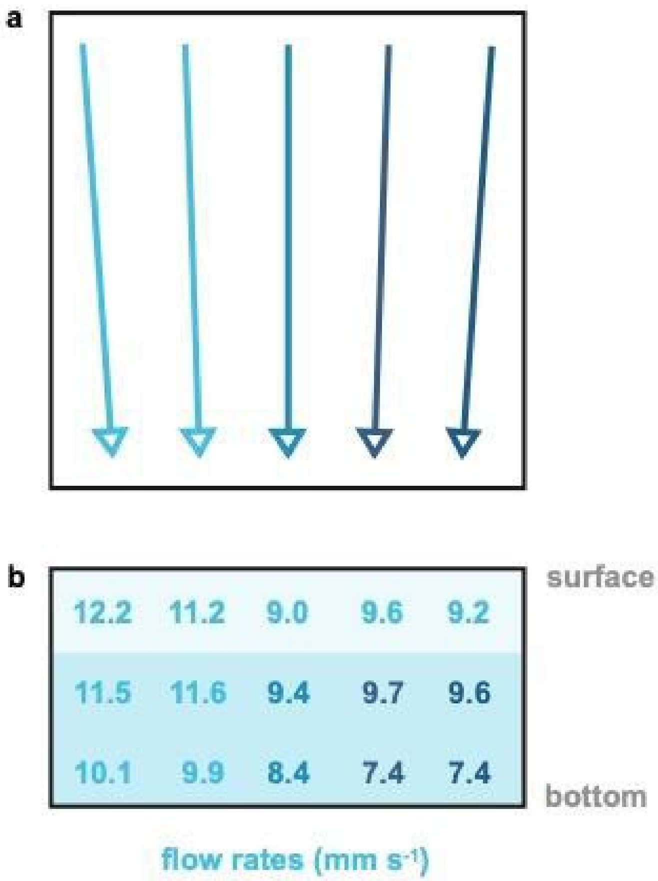

Supplementary Figure 3. Visualization of methylene blue dye tests within the experimental arena shows a laminar yet non-uniform flow field. a) The vectors are color coded according to the mean of the b) cross-sectional flow values (mm s^{-1}) from top to bottom. Each cross-sectional flow value (b) is the mean of five trials. Fish typically occupied the dark shaded area in the bottom two-thirds of the water column and very rarely swam near the surface.

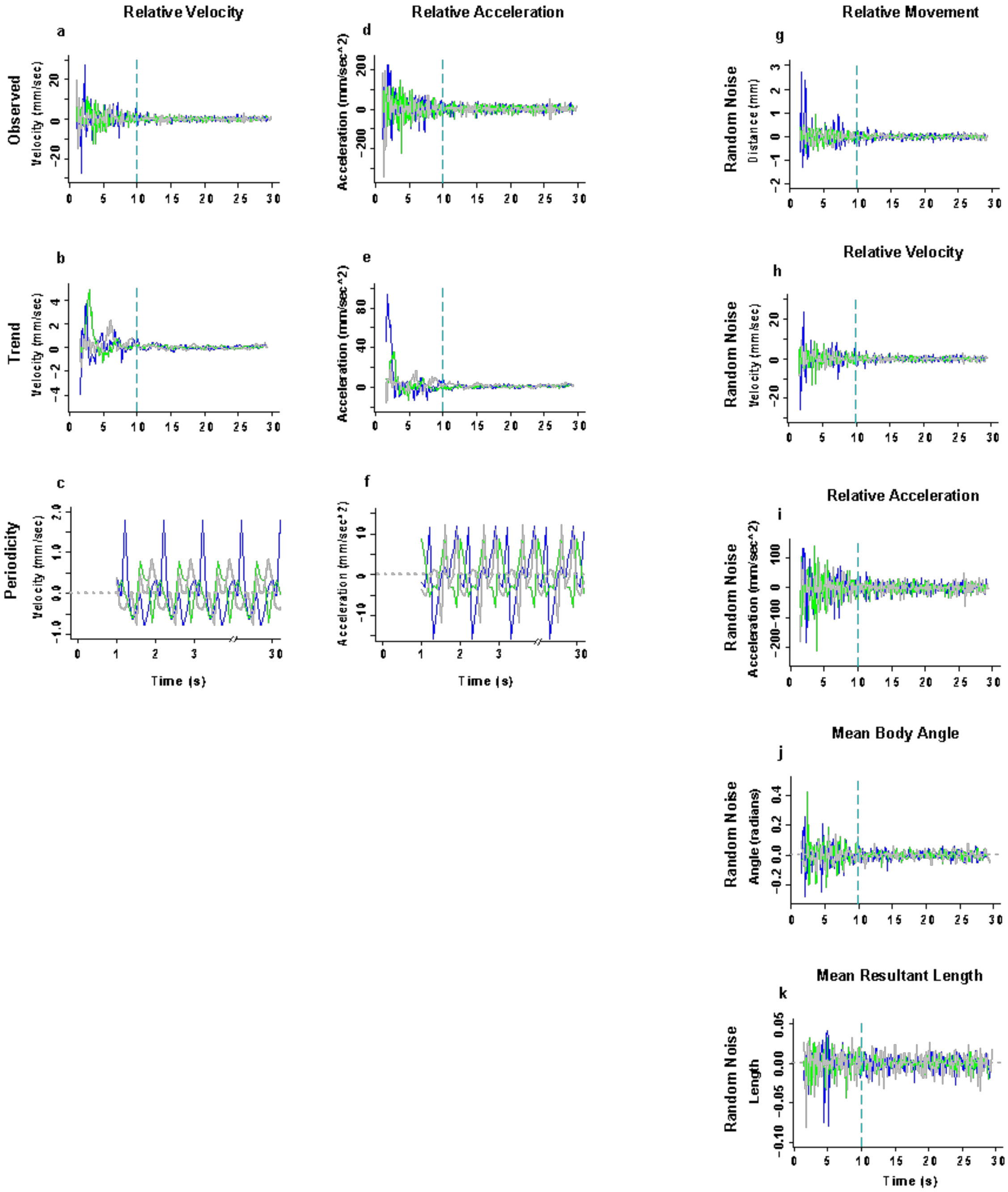

Supplementary Figure 4. The overall trends and periodic fluctuations in the relative velocity and acceleration in rheotaxis behavior differed among treatment groups. Gray = control (n = 248), blue = $CuSO_4$ (n = 204), green = neomycin (n =222). Spectral decomposition of the observed data (a, d) removed the noise (h, i) to reveal the overall underlying trends (b, e) and the periodicity, or recurring fluctuations (c, f) that occurred during any given 1 s of the experiment. The periodicity waveform peaks indicate the average amount (amplitude), number, direction (positive = increasing; negative = decreasing), and order of occurrence for these cyclic fluctuations as a function of unit time (1 s). The overall trends were that there were no differences in relative (b) velocity or (e) acceleration among groups. The periodic fluctuation in relative velocity (c) and acceleration (f) was greatest in $CuSO_4$ treated fish compared to control or neomycin treated fish. The noise for relative movement (g), mean body angle (j), and mean resultant length (k) are shown.

	No Flow Stimulus<br>1-10s<br>*theta, rho,* ang var	Flow Stimulus<br>11-20s<br>*theta, rho,* ang var	Flow Stimulus<br>21-30s<br>*theta, rho,* ang var
Control	135.5°<br>0.084<br>0.916	357.9°<br>0.687<br>0.313	1.7°<br>0.688<br>0.312
CuSO4	84.8 °<br>0.099<br>0.901	10.8°<br>0.530<br>0.470	3.1°<br>0.611<br>0.389
Neomycin	86.7°<br>0.090<br>0.900	6.1°<br>0.656<br>0.344	6.7°<br>0.655<br>0.345

Supplementary Table 1. Grand mean body angle vector parameters. *Theta* = group mean body angle; *rho* = mean length of resultant vector, where 0 = uniform distribution of individual mean body angles, 1 = perfect alignment individual mean body angles; angular variance = 1-*rho*.

	No Flow Stimulus<br>1-10s<br>test stat, p-value	Flow Stimulus<br>11-20s<br>test stat, p-value	Flow Stimulus<br>21-30s<br>test stat, p-value
Control	-0.0595<br>0.9079	0.686<br>**< 0.001**	0.6678<br>**< 0.001**
CuSO4	0.009<br>0.4276	0.5204<br>**< 0.001**	0.6105<br>**< 0.001**
Neomycin	0.0052<br>0.4566	0.6522<br>**< 0.001**	0.6507<br>**< 0.001**

Supplementary Table 2. Rayleigh test of uniformity (V-test) for an expected grand mean body angle, *mu* = 0°. Among all treatments, groups under no flow were not significantly aligned to 0°, whereas groups under flow conditions were significantly aligned to 0°.

	CTL-10s p-value	CTL-20s p-value	Cu-10s p-value	Cu-20s p-value	Neo-10s p-value	Neo-20s p-value
CTL-10s test stat	-	0.4747	**2.58e-05**	0.119	**0.0357**	>0.10
CTL-20s test stat	1.49	-	**7.041e-05**	**0.0508**	0.07419	0.4502
Cu-10s test stat	21.128	19.122	-	0.04975	0.05861	**0.0090**
Cu-20s test stat	4.2573	5.9597	6.0014	-	0.311	0.1921
Neo-10s test stat	6.6655	5.2022	5.6737	2.3357	-	0.7364
Neo-20s test stat	0.1251	1.5961	9.4268	3.3000	0.61196	-

Supplementary Table 3. Watson Wheeler test for differences in either grand mean body angle or the distribution of the individual mean angles (*the test does not specify*) among treatment groups under flow conditions.

GLMM	Estimate	Std. Error	df	t value	Pr(>|t|)
Stimulus (No flow v Flow 10s)	1.14950	0.10746	1390	10.697	**< 2e-16 *** **
Stimulus (No flow v Flow 20s)	1.62296	0.10746	1390	15.102	**< 2e-16 *** **
Treatment (Control v $CuSO_4$)	0.05485	0.11893	2068	0.461	0.64473
Treatment (Control v Neomycin)	0.04574	0.11507	2068	0.398	0.69103
Stim*Treat (CTL-10s v $CuSO_4$-10s)	-0.34246	0.16277	1390	-2.104	**0.03556 ***
Stim*Treat (CTL-20s v $CuSO_4$-20s)	-0.45260	0.16277	1390	-2.781	**0.00550 ****
Stim*Treat (CTL-10s v Neo-10s)	-0.29562	0.15749	1390	-1.877	**0.06072 .**
Stim*Treat (CTL-20s v Neo-20s)	-0.36519	0.15749	1390	-2.319	**0.02055 ***

ANOVA (III)	Sum Sq	Mean Sq	NumDF	DenDF	F value	Pr(>F)
Stimulus	660.88	330.44	2	1390	216.7689	**< 2.2e-16 *** **
Treatment	29.83	14.92	2	695	9.7842	**6.449e-05 *** **
Stim*Treat	15.40	3.85	4	1390	2.5249	**0.03926 ***

Supplementary Table 4. Generalized Linear Mixed Model with Satterthwaite's method of testing for differences among treatments in the mean duration of rheotaxis events. Type III ANOVA yielded significance values for fixed effects and interactions because the LME4 package in R does not identify them in its output. Significance codes: '***' 0.001, '**' 0.01, '*' 0.05, '.' 0.1

GLMM	Estimate	Std. Error	df	t value	Pr(>|t|)
Stimulus (No flow v Flow 10s)	2.65530	0.15538	1390	17.089	**< 2e-16 *** **
Stimulus (No flow v Flow 20s)	2.91667	0.15538	1390	18.771	**< 2e-16 *** **
Treatment (Control v $CuSO_4$)	-0.52696	0.21170	1614	-2.489	**0.0129 * **
Treatment (Control v Neomycin)	0.46123	0.20484	1614	2.252	**0.0245 * **
Stim*Treat (CTL-10s v $CuSO_4$-10s)	-0.57687	0.23535	1390	-2.451	**0.0144 * **
Stim*Treat (CTL-20s v $CuSO_4$-20s)	0.05882	0.23535	1390	0.250	0.8027
Stim*Treat (CTL-10s v Neo-10s)	0.09687	0.22772	1390	0.425	0.6706
Stim*Treat (CTL-20s v Neo-20s)	0.05290	0.22772	1390	0.232	0.8163

ANOVA (III)	Sum Sq	Mean Sq	NumDF	DenDF	F value	Pr(>F)
Stimulus	3488.9	1744.46	2	1390	547.3568	**< 2.2e-16 *** **
Treatment	167.1	83.57	2	695	26.2224	**1.049e-11 *** **
Stim*Treat	40.0	9.99	4	1390	3.1356	**0.01403 * **

Supplementary Table 5. Generalized Linear Mixed Model with Satterthwaite's method of testing for differences among treatments in the mean number of rheotaxis events. Type III ANOVA yielded significance values for fixed effects and interactions because the LME4 package in R does not identify them in its output. Significance codes: '***' 0.001, '**' 0.01, '*' 0.05, '.' 0.1

GLMM	Estimate	Std. Error	df	t value	Pr(>|t|)
Stimulus (No flow v Flow 10s)	0.70177	0.16338	1721	4.295	**1.84e-05 *****
Stimulus (No flow v Flow 20s)	0.65449	0.16338	1721	4.006	**6.44e-05 *****
Treatment (Control v $CuSO_4$)	0.18141	0.11436	695	1.586	0.11313
Treatment (Control v Neomycin)	0.30691	0.11102	706	2.764	**0.00585 ****

ANOVA (III)	Sum Sq	Mean Sq	NumDF	DenDF	F value	Pr(>F)
Stimulus	243.652	81.037	2	1565	38.8494	**< 2.2e-16 *****
Treatment	8.117	3.796	2	699	3.8913	**0.02086 ***

Supplementary Table 6. Generalized Linear Mixed Model with Satterthwaite's method of testing for differences among treatments in the total distance travelled during rheotaxis events. GLMM model had no interaction between stimulus and treatment. Type III ANOVA yielded significance values for fixed effects because the LME4 package in R does not identify them in its output. Significance codes: '***' 0.001, '**' 0.01, '*' 0.05, '.' 0.1

GLMM	Estimate	Std. Error	df	t value	Pr(>|t|)
Stimulus (No flow v Flow 10s)	8.460e+00	3.333e-01	2.085e+03	25.386	**< 2e-16 *** **
Stimulus (No flow v Flow 20s)	1.762e+01	3.333e-01	2.085e+03	52.882	**< 2e-16 *** **
Treatment (Control v $CuSO_4$)	-1.384e+00	3.569e-01	2.085e+03	-3.877	**0.000109 *** **
Treatment (Control v Neomycin)	8.802e-03	2.085e+03	2.085e+03	0.025	0.979669
Stim*Treat (CTL-10s v $CuSO_4$-10s)	.904e-01	5.048e-01	2.085e+03	0.972	0.331412
Stim*Treat (CTL-20s v $CuSO_4$-20s)	1.015e+00	5.048e-01	2.085e+03	2.011	**0.044503 * **
Stim*Treat (CTL-10s v Neo-10s)	-4.100e-02	4.884e-01	2.085e+03	-0.084	0.933106
Stim*Treat (CTL-20s v Neo-20s)	8.078e-02	4.884e-01	2.085e+03	0.165	0.868645

ANOVA (III)	Sum Sq	Mean Sq	NumDF	DenDF	F value	Pr(>F)
Stimulus	112476	56238	2	2089	3834.664	**< 2.2e-16 *** **
Treatment	345	173	2	2089	11.764	**8.306e-06 *** **

Supplementary Table 7. Generalized Linear Mixed Model with Satterthwaite's method of testing for differences among treatments in the mean latency to the onset of first rheotaxis event. GLMM model had no interaction between stimulus and treatment. Type III ANOVA yielded significance values for fixed effects and interactions because the LME4 package in R does not identify them in its output. Significance codes: '***' 0.001, '**' 0.01, '*' 0.05, '.' 0.1

GLM	Estimate	Std. Error	t value	Pr(>|t|)
Treatment (Control v $CuSO_4$)	31.9668	4.4820	7.132	**1.21e-12 *****
Treatment (Control v Neomycin)	14.6849	4.0870	3.593	**0.000332 *****
ROI (Back v Center)	4.6943	4.7014	0.998	0.318122
ROI (Back v Front)	-6.3503	4.7014	-1.351	0.176878
ROI (Back v Left)	-13.8344	4.7014	-2.943	**0.003278 ****
ROI (Back v Right)	7.7325	4.7014	-1.645	0.100126
ROI*Treat (CTL-Back v $CuSO_4$-Center)	10.2432	6.3386	1.616	0.106188
ROI*Treat (CTL-Back v Neo-Center)	733.35	5.7799	3.287	**0.001023 ****
ROI*Treat (CTL-Back v $CuSO_4$-Front)	18.9995	6.3386	-4.265	**2.05e-05 *****
ROI*Treat (CTL-Back v Neo-Front)	-27.0351	5.7799	0.146	0.884161
ROI*Treat (CTL-Back v $CuSO_4$-Left)	-23.8271	6.3386	-3.759	**0.000174 *****
ROI*Treat (CTL-Back v Neo-Left)	-13.3969	5.7799	-2.318	**0.020519 ***
ROI*Treat (CTL-Back v $CuSO_4$-Right)	-21.5175	6.3386	-3.395	**0.000695 *****
ROI*Treat (CTL-Back v Neo-Right)	-10.7854	5.7799	-1.866	**0.062127 .**

ANOVA (III)	Sum Sq	Mean Sq	Df	F value	Pr(>F)
Treatment	172565	86283	2	49.727	**< 2.2e-16 *****
ROI	778750	194687	4	112.204	**< 2.2e-16 *****
Treatment*ROI	135133	16892	8	9.735	**1.93e-13 *****

Supplementary Table 8. Generalized Linear Model tests for differences in the two-dimensional X-Y spatial use among treatments. GLM model included fixed effects of stimulus and treatment, and the effect of the interaction between stimulus and treatment. ANOVA yielded significance values for fixed effects and interactions because the LME4 package in R does not identify them in its output. Significance codes: '***' 0.001, '**' 0.01, '*' 0.05, '.' 0.1

	Control	Control	Control	$CuSO_4$	$CuSO_4$	$CuSO_4$	Cu-Ctl	Cu-Ctl	Neomycin	Neomycin	Neomycin	Neo-Ctl	Neo-Ctl
Variable	Min	Max	Range	Min	Max	Range	ΔRange	ΔRange (factor)	Min	Max	Range	ΔRange	ΔRange (factor)
mov	-0.0431	0.0583	0.1014	-0.0724	0.1547	0.2270	0.1257	**2.2400**	-0.0414	0.0441	0.0855	-0.0158	*0.8437*
vel	-0.7762	0.8416	1.6178	-0.7991	1.7910	2.5900	0.9723	**1.6010**	-0.7129	0.7997	1.5126	-0.1051	*0.9350*
acc	-11.8950	12.3300	24.2250	-15.8962	12.1951	28.0914	3.8664	**1.1596**	-8.1738	9.7733	17.9471	-6.2779	*0.7409*
angle	-0.0158	0.0107	0.0265	-0.0087	0.0145	0.0231	-0.0034	*0.8729*	-0.0162	0.0256	0.0418	0.0153	**1.5766**
res L	-0.0052	0.0075	0.0127	-0.0038	0.0023	0.0061	-0.0066	*0.4832*	-0.0026	0.0028	0.0054	-0.0074	*0.4219*

Supplementary Table 9 (Based on Fig. 7). Lateral line ablation by drug treatment (Cu, Neo) shifts the range in overall amplitude of linear (*mov, vel, acc*) and angular (*angle, res L*) seasonality data relative to those of the control (Ctl) group. Movement parameters in which the amplitude increased in treatment fish relative to controls are indicated in **bold**, whereas those that decreased are in *italics*.

		Control	$CuSO_4$	Cu-Ctl	Cu-Ctl	Neomycin	Neo-Ctl	Neo-Ctl	Control	$CuSO_4$	Cu-Ctl	Cu-Ctl	Neomycin	Neo-Ctl	Neo-Ctl
Variable	Dominant Peak	Freq (1/s)	Freq	ΔFreq (1/s)	Net Shift Freq 1-3	Freq	ΔFreq (1/s)	Net Shift Freq 1-3	Power	Power	ΔPower (factor)	Net Shift Pwr 1-3	Power	ΔPower (factor)	Net Shift Pwr 1-3
mov	1st	0.1567	0.1528	*-0.0039*	*down*	0.2118	*0.0551*	*down*	0.0169	0.0448	**2.6475**	**up**	0.0337	**1.9964**	**up**
mov	2nd	0.0267	0.1042	*0.0775*	-	0.0139	*-0.0128*	-	0.0101	0.0326	**3.2379**	-	0.0139	**1.3814**	-
mov	3rd	0.2533	0.0208	*-0.2325*	-	0.1632	*-0.0901*	-	0.0082	0.0287	**3.5016**	-	0.0129	**1.5777**	-
vel	1st	0.2533	0.1563	*-0.0971*	*down*	0.2118	*-0.0415*	*down*	1.8444	3.1181	**1.6906**	**up**	6.9068	**3.7447**	**up**
vel	2nd	1.8444	0.2604	*-1.5840*	-	0.4792	*-1.3652*	-	1.5272	2.5076	**1.6419**	-	1.4930	**0.9776**	-
vel	3rd	0.3100	0.2118	*-0.0982*	-	0.1632	*-0.1468*	-	0.7717	1.9820	**2.5683**	-	1.3884	**1.7991**	-
acc	1st	0.2500	0.2604	*0.0104*	*down*	0.2118	*-0.0382*	*down*	363.7884	502.2213	**1.3805**	**up**	938.4990	**2.5798**	**up**
acc	2nd	0.4800	0.3056	*-0.1744*	-	0.4410	*-0.0390*	-	244.0334	402.0855	**1.6477**	-	393.4430	**1.6123**	-
acc	3rd	0.3100	0.1458	*-0.1642*	-	0.2847	*-0.0253*	-	204.4871	304.1066	**1.4872**	-	346.3958	**1.6940**	-
angle	1st	0.0400	0.0382	**-0.0018**	**up**	0.0174	**-0.0226**	**up**	0.0029	0.0042	**1.4571**	**up**	0.0040	**1.3874**	**up**
angle	2nd	0.0700	0.1528	**0.0828**	-	0.1250	**0.0550**	-	0.0026	0.0014	**0.5371**	-	0.0028	**1.0712**	-
angle	3rd	0.1167	0.2083	**0.0917**	-	0.1944	**0.0778**	-	0.0009	0.0009	**1.0510**	-	0.0014	**1.6840**	-
res L	1st	0.0600	0.0313	*-0.0288*	*down*	0.1319	**0.0719**	**up**	0.0002	0.0002	**1.3167**	**up**	0.0001	*0.4215*	*down*
res L	2nd	0.3433	0.0625	*-0.2808*	-	0.2396	**-0.1038**	-	0.0001	0.0001	**1.7516**	-	0.0000	*0.7071*	-
res L	3rd	0.1667	0.1319	*-0.0347*	-	0.4097	**0.2431**	-	0.0001	0.0001	**1.3909**	-	0.0000	*0.6849*	-

Supplementary Table 10 (based on Fig. 8). The primary, secondary, and tertiary dominant frequencies (*Freq*) of the linear (*mov, vel, acc*) and angular (*angle, res L*) movement power spectra shift up or down (*±ΔFreq*) in each of the drug treatments (Cu, Neo) compared to the control (Ctl) group. The peak power (*Pwr*) for all dominant frequencies increases (*+ΔPwr*) in nearly all cases. Values for the net shift in frequency and power were determined by adding the values of the +/- relative shift (ΔFrequency, ΔPower) of all three dominant frequencies and generalizing the overall shift in frequency and power as "up" or "down". The trend of CuSO4 and neomycin ablation results in net downshifts in the frequency and net upshift in power of relative movement, velocity and acceleration and a net upshift in the frequency and power of the mean body angle. However, CuSO4 ablation results in a net downshift in the frequency and net upshift in power, whereas neomycin results in a net upshift in the frequency and net downshift in power. Movement parameters in which the amplitude increased in treatment fish relative to controls are indicated in **bold**, whereas those that decreased are in *italics*.

		Control	Control	$CuSO_4$	$CuSO_4$	Neomycin	Neomycin
Linear parameter reference	Angular parameter CCF	Lag: (+/-) before, after	Heading: right, left Variance: more, less	Lag: (+/-) before, after	Heading: right, left Variance: more, less	Lag: (+/-) before, after	Heading: right, left Variance: more, less
movement	body angle	before	left	**after**	**left**	*after*	*right*
movement	resultant length	before	less	**before**	**less**	*after*	*less*
velocity	body angle	simultaneous	right	*after*	*right*	**after**	**left**
velocity	resultant length	before	less	*after*	*less*	**before**	**less**
acceleration	body angle	**after**	**left**	*after*	*right*	**after**	**left**
acceleration	resultant length	**before**	**less**	*after*	*less*	**before**	**less**

Supplementary Table 11 (based on Fig. 9). The method, and perhaps mechanism, of lateral line ablation by. $CuSO_4$ and neomycin results in two distinct type of rheotaxis kinematics that are different from control fish. Each method has the opposite effect on the linear and angular movement parameters during rheotaxis and neither matches the phenotype of fish with an intact lateral line. Behavioral phenotypes in which an above average increase in the indicated linear variable was strongly correlated with a leftward heading that will occurr later and a decrease in angular variance that occurred previously are indicated in **bold**. Phenotypes in which an above average increase in the indicated linear variable was strongly correlated with a rightward heading and a decrease in angular variance that will occur later are indicated in *italics*.
