# Supplementary material for: Lateral line ablation by ototoxic compounds results in distinct rheotaxis profiles in larval zebrafish
Source: Commun Biol. 2023 Jan 21;6:84. doi: 10.1038/s42003-023-04449-2 (PMC9867717; doi:10.1038/s42003-023-04449-2)
Supplement: Supplementary file 3 — Description of Additional Supplementary Files [file 42003_2023_4449_MOESM3_ESM.pdf]

Description of Additional Supplementary Files

File name: Supplementary Movie 1
Description: Untreated larval zebrafish (control) in the microflume arena before stimulus onset (10 sec) and during water flow (20 sec) performing rheotaxis as defined in Supplementary Figure 1.

File name: Supplementary Movie 2
Description: $CuSO_4$ treated larval zebrafish in the microflume arena before stimulus onset (10 sec) and during water flow (20 sec) performing rheotaxis as defined in Supplementary Figure 1.

File name: Supplementary Movie 3
Description: Neomycin treated larval zebrafish in the microflume arena before stimulus onset (10 sec) and during water flow (20 sec) performing rheotaxis as defined in Supplementary Figure 1.
