# Supplementary material for: Lateral line ablation by ototoxic compounds results in distinct rheotaxis profiles in larval zebrafish
Source: Commun Biol. 2023 Jan 21;6:84. doi: 10.1038/s42003-023-04449-2 (PMC9867717; doi:10.1038/s42003-023-04449-2)
Supplement: Supplementary file 7 — Reporting Summary [file 42003_2023_4449_MOESM7_ESM.pdf]

nature portfolio

Corresponding author(s): Kyle Newton, PhD
Lavinia Sheets, PhD

Last updated by author(s): Nov 30, 2022

Reporting Summary

Nature Portfolio wishes to improve the reproducibility of the work that we publish. This form provides structure for consistency and transparency in reporting. For further information on Nature Portfolio policies, see our Editorial Policies and the Editorial Policy Checklist.

Statistics

For all statistical analyses, confirm that the following items are present in the figure legend, table legend, main text, or Methods section.

n/a	Confirmed	
☐	☒	The exact sample size (n) for each experimental group/condition, given as a discrete number and unit of measurement
☐	☒	A statement on whether measurements were taken from distinct samples or whether the same sample was measured repeatedly
☐	☒	The statistical test(s) used AND whether they are one- or two-sided<br>*Only common tests should be described solely by name; describe more complex techniques in the Methods section.*
☒	☐	A description of all covariates tested
☐	☒	A description of any assumptions or corrections, such as tests of normality and adjustment for multiple comparisons
☐	☒	A full description of the statistical parameters including central tendency (e.g. means) or other basic estimates (e.g. regression coefficient) AND variation (e.g. standard deviation) or associated estimates of uncertainty (e.g. confidence intervals)
☐	☒	For null hypothesis testing, the test statistic (e.g. F, t, r) with confidence intervals, effect sizes, degrees of freedom and P value noted<br>*Give P values as exact values whenever suitable.*
☒	☐	For Bayesian analysis, information on the choice of priors and Markov chain Monte Carlo settings
☒	☐	For hierarchical and complex designs, identification of the appropriate level for tests and full reporting of outcomes
☒	☐	Estimates of effect sizes (e.g. Cohen's d, Pearson's r), indicating how they were calculated

Our web collection on statistics for biologists contains articles on many of the points above.

Software and code

Policy information about availability of computer code

Data collection	Our GPU equipment required the installation of Tensorflow 1.12 with the NVIDIA CUDA package prior to installing multi-animal DeepLabCut2.2b8 (maDLC; 42Mathis et al. 2018, 43Nath et al. 2019), Python 3.6, and all dependencies in an Anaconda virtual environment according to (https://github.com/DeepLabCut/DeepLabCut/blob/master/docs/installation.md). SimBAxTF-development version 68 (44Nilsson et al. 2020), Python 3.6, Git, FFmpeg, and all necessary dependencies were installed in a separate Anaconda virtual environment: (https://github.com/sgoldenlab/simba/blob/master/docs/installation.md).
Data analysis	The R code generated for the analyses during the current study are available in the Open Science Framework repository, https://osf.io/rvyfz/

For manuscripts utilizing custom algorithms or software that are central to the research but not yet described in published literature, software must be made available to editors and reviewers. We strongly encourage code deposition in a community repository (e.g. GitHub). See the Nature Portfolio guidelines for submitting code & software for further information.

Data

Policy information about availability of data

All manuscripts must include a data availability statement. This statement should provide the following information, where applicable:

- Accession codes, unique identifiers, or web links for publicly available datasets
- A description of any restrictions on data availability
- For clinical datasets or third party data, please ensure that the statement adheres to our policy

The datasets generated during and/or analyzed during the current study are available in the Open Science Framework repository, https://osf.io/rvyfz/

Field-specific reporting

Please select the one below that is the best fit for your research. If you are not sure, read the appropriate sections before making your selection.

☒ Life sciences ☐ Behavioural & social sciences ☐ Ecological, evolutionary & environmental sciences

For a reference copy of the document with all sections, see nature.com/documents/nr-reporting-summary-flat.pdf

Life sciences study design

All studies must disclose on these points even when the disclosure is negative.

Sample size	N= 248 (controls), 204 (CuSO4), 222 (neomycin). Sample size was not pre-determined but we chose them to be commensurate with a pilot study that tested fish rheotaxis in groups of five individuals. We reasoned that similar sample sizes would facilitate future comparison of rheotaxis behavior between fish tested alone and in groups.
Data exclusions	Only missing data (<4%) were excluded from the analyses. In such rare cases the tracking of individuals was not possible because the software model, despite being optimized to maximum efficacy, could not always identify the body parts of the transparent larvae under the low contrast IR illumination. These data were recorded as zeros but not included to avoid skewing the central tendency computations (e.g. means, SE).
Replication	Experiments were replicated 18 times to ensure data reproducibility and reduced variance among individuals and groups
Randomization	Individual animals were allocated to groups by combining all larvae into a single large beaker then randomly selecting individuals with a transfer pipette and placing them into treatment groups in random order.
Blinding	Data acquisition, animal tracking, and behavioral annotation analyses were done impartially by the computer using DeepLabCut and SimBA software so it was not necessary for the investigator to be blind to group allocation.

Reporting for specific materials, systems and methods

We require information from authors about some types of materials, experimental systems and methods used in many studies. Here, indicate whether each material, system or method listed is relevant to your study. If you are not sure if a list item applies to your research, read the appropriate section before selecting a response.

Materials & experimental systems

n/a	Involved in the study	
☐	☒	Antibodies
☒	☐	Eukaryotic cell lines
☒	☐	Palaeontology and archaeology
☐	☒	Animals and other organisms
☒	☐	Human research participants
☒	☐	Clinical data
☒	☐	Dual use research of concern

Methods

n/a	Involved in the study	
☒	☐	ChIP-seq
☒	☐	Flow cytometry
☒	☐	MRI-based neuroimaging

Antibodies

Antibodies used	Otoferlin (HCS-1, Developmental Studies Hybridoma Bank, mouse IgG2a, 1:500), Calbindin D28k (Synaptic Systems, mouse Ig1, 1:1000)
Validation	Antibody information and validation can be found on the DSHB website (https://dshb.biology.uiowa.edu/HCS-1)and Synaptic Systems website (https://sysy.com/product/214011)

Animals and other organisms

Policy information about studies involving animals; ARRIVE guidelines recommended for reporting animal research

Laboratory animals	Larval zebrafish, AB* strain, 6-7 days post fertilization, sex indeterminate i.e. no sex differences in zebrafish at this age
Wild animals	*Provide details on animals observed in or captured in the field; report species, sex and age where possible. Describe how animals were caught and transported and what happened to captive animals after the study (if killed, explain why and describe method; if released, say where and when) OR state that the study did not involve wild animals.*

Field-collected samples	*For laboratory work with field-collected samples, describe all relevant parameters such as housing, maintenance, temperature, photoperiod and end-of-experiment protocol OR state that the study did not involve samples collected from the field.*
Ethics oversight	This study was performed with the approval of the Institutional Animal Care and Use Committee of Washington University School of Medicine in St. Louis (Protocol number: 20-0158) and in accordance with NIH guidelines for use of zebrafish.

Note that full information on the approval of the study protocol must also be provided in the manuscript.
